# The Utility of Next-Generation Sequencing in Gene Discovery for Mutation-Negative Patients with Rett Syndrome

**DOI:** 10.3389/fncel.2015.00266

**Published:** 2015-07-14

**Authors:** Wendy Anne Gold, John Christodoulou

**Affiliations:** ^1^Western Sydney Genetics Program, New South Wales Centre for Rett Syndrome Research, Children’s Hospital at Westmead, Sydney, NSW, Australia; ^2^Discipline of Paediatrics and Child Health, University of Sydney, Sydney, NSW, Australia; ^3^Discipline of Genetic Medicine, Sydney Medical School, University of Sydney, Sydney, NSW, Australia

**Keywords:** Rett syndrome, mutation, massively parallel sequencing, next-generation sequencing, intellectual disability

## Abstract

Rett syndrome (RTT) is a rare, severe disorder of neuronal plasticity that predominantly affects girls. Girls with RTT usually appear asymptomatic in the first 6–18 months of life, but gradually develop severe motor, cognitive, and behavioral abnormalities that persist for life. A predominance of neuronal and synaptic dysfunction, with altered excitatory–inhibitory neuronal synaptic transmission and synaptic plasticity, are overarching features of RTT in children and in mouse models. Over 90% of patients with classical RTT have mutations in the X-linked methyl-CpG-binding (*MECP2*) gene, while other genes, including cyclin-dependent kinase-like 5 (*CDKL5*), Forkhead box protein G1 (*FOXG1*), myocyte-specific enhancer factor 2C (*MEF2C*), and transcription factor 4 (*TCF4*), have been associated with phenotypes overlapping with RTT. However, there remain a proportion of patients who carry a clinical diagnosis of RTT, but who are mutation negative. In recent years, next-generation sequencing technologies have revolutionized approaches to genetic studies, making whole-exome and even whole-genome sequencing possible strategies for the detection of rare and *de novo* mutations, aiding the discovery of novel disease genes. Here, we review the recent progress that is emerging in identifying pathogenic variations, specifically from exome sequencing in RTT patients, and emphasize the need for the use of this technology to identify known and new disease genes in RTT patients.

## Introduction

Rett syndrome (RTT) is a pervasive disorder of neuronal plasticity, characterized by an apparent normal early development, followed by a stagnation and regression of development, leading to loss of purposeful hand movements, reduced brain and head growth, physical disabilities, language and learning deficits, seizures, and intellectual disability (Rett, 1966; Hagberg et al., 1983). Most patients first exhibit symptoms between 6 and 18 months of age and display the hallmark clinical course of progressive loss of cognitive function and fine and gross motor skills, and abnormal social-cognitive development (Williamson and Christodoulou, 2006).

In most cases, RTT is caused by *de novo* mutations in the X-linked methyl-CpG-binding (*MECP2*) gene (Christodoulou and Weaving, 2003), resulting in the disruption of the molecular functions of MeCP2. MeCP2 is predominantly expressed in the brain and particularly in post-mitotic neurons, where its level of expression correlates with the maturation of the central nervous system (Pelka et al., 2005). The distinct neurological phenotype of RTT patients demonstrates that MeCP2 regulation is essential for normal neuronal and brain development and function (Swanberg et al., 2009). The broad clinical phenotype of RTT may make clinical diagnosis a challenge, particularly in mutation-negative patients where a definitive genetic result cannot be used. However, consensus criteria have been established that distinguish RTT patients into the individual classifications of “classic” or “typical” RTT and the “atypical” or “variant” forms of RTT (Neul et al., 2010).

Despite severe neurological and associated behavioral abnormalities observed in RTT, there are no overt differences in the gross structure of the brains in RTT patients (Chahrour and Zoghbi, 2007). However, the number of studies showing structural and morphological synaptic defects (Armstrong et al., 1995, 1998; Bauman et al., 1995; Kishi and Macklis, 2004; Belichenko et al., 2009), reduced synapse number (Belichenko et al., 2009), and synaptic and circuitry deficits (Fukuda et al., 2005; Asaka et al., 2006; Moretti et al., 2006; Guy et al., 2007; Zhang et al., 2008; Lonetti et al., 2010) highlights the synaptic dysfunction associated with RTT.

**Figure 1:**
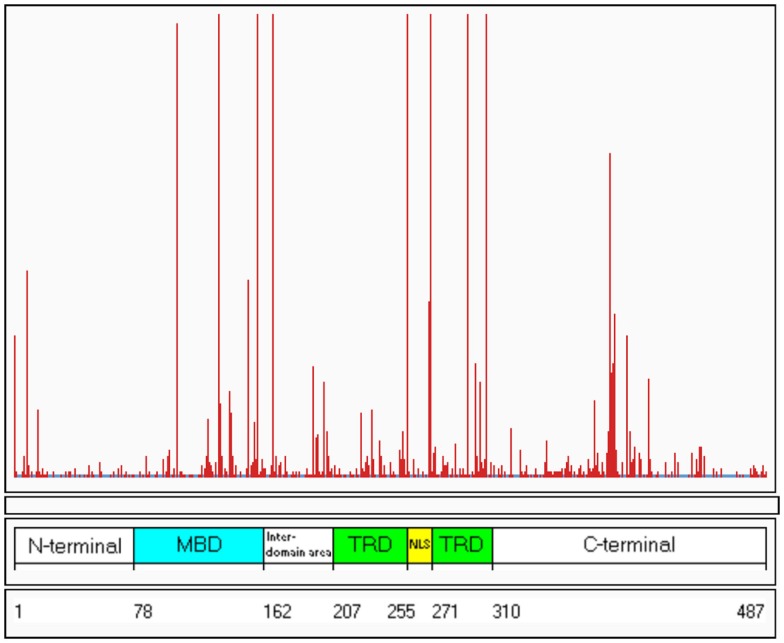
**Distribution of known mutations along the *MECP2* gene**. The red lines indicate the frequency of the mutations. The numbers 1–487 refer to the amino acids along the MeCP2 polypeptide. MDB, methyl-binding domain; TRD, transcription repression domain (RettBASE: http://mecp2.chw.edu.au) (Williamson and Christodoulou, 2006).

To date, over 800 pathogenic mutations have been detected within the *MECP2* gene (RettBASE; Christodoulou et al., 2003)^1^. These mutations include a range of missense, nonsense, frameshift, and in-frame insertions or deletions, as well as large deletions spanning whole exons or even the entire gene. Approximately 80–85% of the known MECP2 mutations are localized within the methyl-binding domain (MBD), transcription repression domain (TRD), and C-terminal domain, creating “hot-spot” areas of mutations (Figure 1).

The *MECP2* gene is alternatively spliced, generating two isoforms *MECP2E1* (previously referred to as *MECP2B* or *MECP2*α) and *MECP2E2* (previously referred to as *MeCP2A* or *MECP2*β) (Kriaucionis and Bird, 2004; Mnatzakanian et al., 2004). The *MeCP2E1* isoform uses the translation start site (ATG) in exon 1 and comprises exons 1, 3, and 4, and is the predominant isoform in the central nervous system, whereas the *MeCP2E2* isoform uses a translation start site in exon 2 and comprises exons 2, 3, and 4. Both isoforms share MBD, TRD, and C-terminal domains, characteristic of *MECP2*.

Although the majority of RTT patients have mutations in the *MECP2* gene (Neul et al., 2008), approximately 8% of classical RTT and 42% of variant RTT patients are *MECP2* mutation negative (Percy, 2008). Of this latter group of individuals, some have mutations in other genes, such as cyclin-dependent kinase-like 5 (*CDKL5)*, Forkhead box protein G1 (*FOXG1)*, myocyte-specific enhancer factor 2C (*MEF2C*), and transcription factor 4 (*TF4*) (Evans et al., 2005a; Philippe et al., 2010; Armani et al., 2012). Interestingly, known RTT-causing *MECP2* mutations are found in patients who do not show classical RTT phenotypes (Suter et al., 2014), as well as in patients with PPM-X syndrome (Klauck et al., 2002), and an “Angelman-like syndrome” clinical picture (Watson et al., 2001). Of note, there still remain a subset of patients with a clinical diagnosis of RTT that are mutation negative.

## Implications in Screening RTT Patients for *MECP2* Mutations

Historically, exon 1 of the *MECP2E1* isoform was excluded from sequencing and mutation analysis in RTT patients as it was thought to be a non-coding exon. However, since the discovery of the *MECP2E1* isoform, mutation analysis of exon 1 has been included and to date, a number of mutations involving exon 1 have been reported in RTT patients (Ravn et al., 2005; Bartholdi et al., 2006; Chunshu et al., 2006; Quenard et al., 2006; Saxena et al., 2006; Saunders et al., 2009; Gianakopoulos et al., 2012). Initially, mutations in exon 1 were suggested to be rare in RTT patients as detection rates were found to be between 0.03 and 1% of tested RTT patients (Amir et al., 2005; Evans et al., 2005b; Quenard et al., 2006). It has also been suggested that RTT patients with mutations in exon 1 have a more severe phenotype than patients without exon 1 mutations (Bartholdi et al., 2006).

To date, no mutations have been reported in exon 2 encoding for the *MeCP2E2* isoform. Mouse studies showing the maternally transferred *Mecp2e2* null allele resulted in reduced embryonic viability, suggesting a likely explanation for the absence of exon 2-specific mutations in RTT patients. Interestingly, some patients with atypical RTT phenotypes, who are *MECP2* mutation negative, show abnormal expression of both *MECP2* isoforms (Petel-Galil et al., 2006), suggesting that other genes may regulate *MECP2* expression.

Recent developments in massively parallel DNA sequencing (also known as next-generation sequencing; NGS) now allow for cost-efficient sequencing of entire exomes (whole-exome sequence; WES) or even the complete genome (whole-genome sequencing; WGS), enabling the efficient identification of novel variants in known disease genes, as well as facilitating the discovery of novel disease genes. NGS is emerging as a new tool for the identification of novel genes in complex genetic disorders, such as RTT. To date, a small number of studies have emerged identifying new disease genes associated with RTT. These studies are discussed below and highlight the utility of NGS in identifying new disease genes in RTT patients.

## Recent Studies Using NGS in “RTT” Patients

In 2014, WES unveiled a variation in the gamma-aminobutyric acid receptor delta gene (*GABRD*) in a 12-year-old female patient diagnosed with an RTT-like clinical picture (Okamoto et al., 2014b). The patient had severe intellectual disability, hypotonia, and a stereotypic behavior, “hand gripping,” described as being typical of RTT, bruxism, and exhibited no purposeful hand skills, was unable to walk independently, and could not articulate any meaningful words. She also had short stature, was underweight, and had microcephaly. Her EEG showed bilateral occipital dominant high-voltage slow spike and wave complexes. Her brain CT and MRI were normal. To determine the underlying genetic cause in this patient, WES was performed, which revealed two *de novo* missense variations in adjacent amino acids in the *GABRD* gene (c.498G > A: p.Met166Ile and c.499G > A: p.Asp167Asn) (Okamoto et al., 2014b), making this the first report of a mutation in the *GABRD* gene in a patient with an RTT-like disorder. *GABRD* is located on chromosome 1p36, encodes a subunit of the ligand-gated chloride channel for the major inhibitory neurotransmitter gamma-aminobutyric acid (GABA) (Windpassinger et al., 2002), and is highly expressed in the brain. Based on the knowledge that mutations in GABA_A_ receptor subunit genes and in *GABRD* are associated with genetic epilepsy syndromes (Emberger et al., 2000), and the observation that patients with 1p36 deletion syndrome often have seizures (Rosenfeld et al., 2010), this suggests that the variation observed in the *GABRD* gene is likely to cause increased neuronal excitability. However, these authors did not provide any *in silico* evidence to support pathogenicity of these variations. From our *in silico* predictions, MutationTaster^2^ predicts these mutations to be “disease causing” with a probability of 0.9999 due to amino acid sequence changes in a highly conserved region, which is predicted to affect splicing. In addition, Polyphen^3^ predicts both of them to be “probably damaging” with a score of 0.969 and PROVEAN^4^ predicts them to be “deleterious” with scores of −3.670 and −4.576, respectively (where the cut-off score is −2.5). Regardless of these predictions, it was not determined whether these two variations were in *cis* or *trans* and no functional studies to support the pathogenicity of RTT were reported. Moreover, our interrogation of the literature failed to identify other *GABRD* mutation-positive cases exhibiting phenotypic overlap with RTT. Accordingly for now, the contention of these authors that mutations in *GABRD* could cause RTT must be cast in doubt.

In another recent case report of a 6-year-old Japanese girl initially reported to have an RTT-like phenotype, WES identified a variation in the WD repeat domain 45 (*WDR45*) gene (Okamoto et al., 2014a). The patient’s development was reported to be normal for the first 10 months, after which she developed stereotypic hand-wringing and finger-sucking behavior typical of RTT, and poor eye contact (which is not typical of *MECP2* mutation-positive “classic” RTT patients). She subsequently developed seizures and her EEG showed diffuse spike-wave and polyspike-wave bursts. Her MRI showed delayed myelination and enlarged lateral ventricles. At 5 years of age, she was noted to be dysmorphic (including hypertelorism, epicanthal folds, flat nasal bridge, bilateral low-set ears, downslanting palpebral fissures, short philtrum, high palate, downturned mouth, and micrognathia), had spasticity of lower limbs and bruxism, was unable to walk independently, had intellectual disability, and had no meaningful speech. Array CGH was normal as was molecular testing for RTT, although it was not stipulated which genes were screened or how. WES revealed a heterozygous *de novo* nonsense mutation causing an early termination in the X-linked WD repeat domain 45 (*WDR45*) gene (c.868C > T: p.Gln290*). Mutations of *WDR45* have been reported to cause β-propeller protein-associated neurodegeneration (BPAN), characterized by early intellectual disability, followed by progressive motor and cognitive deterioration with onset in the second to third decade. This particular variation has not been previously reported in the *WDR45* gene and our *in silico* analysis reveals it to be “disease causing” with a high probability of 1, according to MutationTaster and even “deleterious” according to PROVEAN with a score of −5.656, highlighting the severity of this variation and its impact on pathogenicity. Patients with BPAN show a typical picture of brain iron accumulation on MRI, and indeed a subsequent MRI in this patient showed such abnormalities. Stereotypic hand movements, including hand wringing and flapping, have been reported in patients with BPAN (Saitsu et al., 2013) and in fact in another study, 23 BPAN patients were suspected to have atypical RTT (Hayflick et al., 2013), suggesting an overlap in symptoms between these disorders. Again, the authors did not show any functional data to confirm the pathogenicity of this variation and it could be speculated that this variation may not necessarily be contributing to the RTT phenotype of the patient. The careful evaluation of all available clinical and laboratory data in *MECP2* mutation-negative patients with RTT-like symptoms in interpreting genomic sequencing data is essential. In both these cases, a definitive classification of RTT was not noted. Neither girl was reported to demonstrate a period of developmental regression, and thus we can conclude that they did not have the classical form of RTT, but rather an RTT-like phenotype overlapping with BPAN. It can only be assumed that the dysmorphic features are a consequence of the primary genetic disorder, as facial dysmorphism is not a feature of RTT.

Whole-exome sequence has also been used to recently identify a variation in the *CDKL5* gene in a 5-year-old Japanese boy with intractable epilepsy, severe developmental delay, and RTT-like features (Kato et al., 2015). Variations in *CDKL*5 are associated with epileptic encephalopathy (Bahi-Buisson et al., 2008). At the age of 2 months, he contracted a respiratory-syncytial virus and developed non-febrile cluster clonic convulsions. Computed tomography and MRI of the brain and EEG were all normal at that time. He continued to have tonic seizures several times a day and EEG showed sporadic single polyspikes and diffuse irregular polyspikes. At 17 months, motor and developmental delay was evident: he could sit unaided but could not crawl, could not follow with his eyes, speak any meaningful words, or stand unaided. He continued to have intractable seizures and an abnormal EEG. WES revealed variations in the two genes, *CDKL5* and *KCNQ2*. The variation in *CDKL5* was a *de novo* hemizygous mutation in (c.119C > T, p.Ala40Val), which has been previously reported to be responsible for early infantile epileptic encephalopathy (Rosas-Vargas et al., 2008). He was also heterozygous for a paternally inherited previously reported variation in the potassium voltage-gated channel, KQT-like subfamily, member 2 gene (*KCNQ2*) (c.1545G > C, p.Glu515Asp). Mutations in this gene cause benign familial neonatal seizures, and so it seems unlikely that this variation has contributed to this child’s epileptic encephalopathy, but this is not easily testable. CDKL5-related encephalopathy is an X-linked dominant disorder that is characterized by early infantile epileptic encephalopathy that is clinically distinct from RTT (Fehr et al., 2013). These findings highlight the utility of WES in identifying the etiology of the complex infantile seizure disorder in this patient.

Another study, by Grillo and colleagues, explored the prospect of RTT being a complex genetic disorder using WES. They studied two pairs of sisters with pathogenic *MECP2* mutations (the c.1157del32 mutation inherited from their mother in one pair of sisters, and an apparently *de novo MECP2* deletion including exon 3 and part of exon 4, respectively, in the other pair of sisters) (Grillo et al., 2013). Although the sisters shared the same *MECP2* mutation, they were discordant in their clinical severity, which could be explained by differences in skewing of X-chromosome inactivation. Whole-exome sequencing revealed variations in a number of genes involved in oxidative stress, muscle impairment, and intellectual disability and/or autism in the more severely affected individuals, whereas their sisters with a milder RTT phenotype had variations in genes related to the regulation of the immune system. Whether these variants were of functional relevance in these sisters remains an open question, but this study raises the interesting prospect that NGS strategies could potentially unmask genetic modifiers of phenotypic severity in *MECP2* mutation-positive RTT patients, which could in turn point to potential novel therapeutic targets.

Gilissen and colleagues used WGS to identify large deletions in patients with RTT (Gilissen et al., 2014). In one girl with a clinical diagnosis of RTT, an intra-exonic deletion within exon 4 of the *MECP2* gene was identified, which for technical reasons had been missed by Sanger sequencing and MLPA analysis performed in a diagnostic laboratory. In the same study, another patient with RTT-like symptoms was identified to have a single exon deletion in the structural maintenance of chromosomes 1A (*SMC1A*) gene. This patient had some features seen in RTT (severe intellectual disability, microcephaly, stereotypic movements, short stature, and scoliosis), but also had features not at all typical of RTT (facial dysmorphism, cleft palate, cataracts, tapered fingers, and brain MRI abnormalities). Mutations in *SMC1A* cause Cornelia de Lange syndrome, and in retrospect this child’s clinical picture was a good fit for this disorder. These cases highlight the power of WGS to identify sub-exonic deletions in patients with RTT or disorders that may share some features with RTT, and emphasize the potential utility of NGS, in particular WGS, to efficiently identify existing disease genes in patients with complex phenotypes.

## Challenges of NGS and Moving Beyond the Exome

Whole-exome sequence is emerging as an effective approach to identify known and even novel causative genes in patients with RTT or RTT-like phenotypes, offering comprehensive coverage of the majority of the coding regions of the genome. However, there are some limitations in the technology. These include potentially poor coverage of GC rich or repetitive regions, the fact that the target enrichment strategies do not include non-coding regions, such as introns, 3′-UTR and 5′-UTR regions, and the inability to easily detect structural variations, such as inversions, copy number variations, and translocations, although bioinformatics resources are improving the capacity to identify such structural variations (Bellos and Coin, 2014). As WGS costs fall and the analysis of massive genomic sequencing data files improve with time, WGS may prove to be a more efficient and cost-effective option to screen for variations in RTT patients.

This report highlights the emerging utility of NGS for the identification of known and novel genes in patients with RTT and overlapping clinical phenotypes. The real utility in NGS lies particularly in the differential genetic diagnosis for the variant forms of the disorder and the discovery of potential genetic modifiers of phenotypic severity. The utilization of this technology will allow for the definitive diagnosis of RTT patients who are mutation negative and will reveal avenues for future translational research targeting new disease genes. WES may open doors for the discovery of new disease genes in RTT and related phenotypes. However, caution must be exercised to ensure that a clinical diagnosis of classical or variant RTT is robust, and care must be taken to prevent overinterpretation of genomic sequencing data. Before such discoveries could be considered to be genuinely pathogenic in RTT patients robust bioinformatic analyses must be performed, supported by clear *in vitro* and/or *in vivo* evidence of functional perturbation.

## Conflict of Interest Statement

The authors declare that the research was conducted in the absence of any commercial or financial relationships that could be construed as a potential conflict of interest.

## Footnotes

^1^http://mecp2.chw.edu.au

^2^http://www.mutationtaster.org/

^3^http://genetics.bwh.harvard.edu/pph2/

^4^http://provean.jcvi.org/index.php

## References

[B1] Amir R. E. Fang P. Yu Z. Glaze D. G. Percy A. K. Zoghbi H. Y. (2005). Mutations in exon 1 of MECP2 are a rare cause of Rett syndrome. J. Med. Genet. 42, e15 10.1136/jmg.2004.026161 15689438 PMC1735975

[B2] Armani R. Archer H. Clarke A. Vasudevan P. Zweier C. Ho G. (2012). Transcription factor 4 and myocyte enhancer factor 2C mutations are not common causes of Rett syndrome. Am. J. Med. Genet. A 158A, 713–719. 10.1002/ajmg.a.34206 22383159

[B3] Armstrong D. Dunn J. K. Antalffy B. Trivedi R. (1995). Selective dendritic alterations in the cortex of Rett syndrome. J. Neuropathol. Exp. Neurol. 54, 195–201. 10.1097/00005072-199503000-00006 7876888

[B4] Armstrong D. D. Dunn K. Antalffy B. (1998). Decreased dendritic branching in frontal, motor and limbic cortex in Rett syndrome compared with trisomy 21. J. Neuropathol. Exp. Neurol. 57, 1013–1017. 10.1097/00005072-199811000-00003 9825937

[B5] Asaka Y. Jugloff D. G. Zhang L. Eubanks J. H. Fitzsimonds R. M. (2006). Hippocampal synaptic plasticity is impaired in the Mecp2-null mouse model of Rett syndrome. Neurobiol. Dis. 21, 217–227. 10.1016/j.nbd.2005.07.005 16087343

[B6] Bahi-Buisson N. Kaminska A. Boddaert N. Rio M. Afenjar A. Gerard M. (2008). The three stages of epilepsy in patients with CDKL5 mutations. Epilepsia 49, 1027–1037. 10.1111/j.1528-1167.2007.01520.x 18266744

[B7] Bartholdi D. Klein A. Weissert M. Koenig N. Baumer A. Boltshauser E. (2006). Clinical profiles of four patients with Rett syndrome carrying a novel exon 1 mutation or genomic rearrangement in the MECP2 gene. Clin. Genet. 69, 319–326. 10.1111/j.1399-0004.2006.00604.x 16630165

[B8] Bauman M. L. Kemper T. L. Arin D. M. (1995). Microscopic observations of the brain in Rett syndrome. Neuropediatrics 26, 105–108. 10.1055/s-2007-979737 7566446

[B9] Belichenko P. V. Wright E. E. Belichenko N. P. Masliah E. Li H. H. Mobley W. C. (2009). Widespread changes in dendritic and axonal morphology in Mecp2-mutant mouse models of Rett syndrome: evidence for disruption of neuronal networks. J. Comp. Neurol. 514, 240–258. 10.1002/cne.22009 19296534

[B10] Bellos E. Coin L. J. (2014). cnvOffSeq: detecting intergenic copy number variation using off-target exome sequencing data. Bioinformatics 30, i639–i645. 10.1093/bioinformatics/btu475 25161258 PMC4147927

[B11] Chahrour M. Zoghbi H. Y. (2007). The story of Rett syndrome: from clinic to neurobiology. Neuron 56, 422–437. 10.1016/j.neuron.2007.10.001 17988628

[B12] Christodoulou J. Grimm A. Maher T. Bennetts B. (2003). RettBASE: the IRSA MECP2 variation database-a new mutation database in evolution. Hum. Mutat. 21, 466–472. 10.1002/humu.10194 12673788

[B13] Christodoulou J. Weaving L. S. (2003). MECP2 and beyond: phenotype-genotype correlations in Rett syndrome. J. Child Neurol. 18, 669–674. 10.1177/08830738030180100901 14649547

[B14] Chunshu Y. Endoh K. Soutome M. Kawamura R. Kubota T. (2006). A patient with classic Rett syndrome with a novel mutation in MECP2 exon 1. Clin. Genet. 70, 530–531. 10.1111/j.1399-0004.2006.00712.x 17101000

[B15] Emberger W. Windpassinger C. Petek E. Kroisel P. M. Wagner K. (2000). Assignment of the human GABAA receptor delta-subunit gene (GABRD) to chromosome band 1p36.3 distal to marker NIB1364 by radiation hybrid mapping. Cytogenet. Cell Genet. 89, 281–282. 10.1159/000015636 10965146

[B16] Evans J. C. Archer H. L. Colley J. P. Ravn K. Nielsen J. B. Kerr A. (2005a). Early onset seizures and Rett-like features associated with mutations in CDKL5. Eur. J. Hum. Genet. 13, 1113–1120. 10.1038/sj.ejhg.5201451 16015284

[B17] Evans J. C. Archer H. L. Whatley S. D. Kerr A. Clarke A. Butler R. (2005b). Variation in exon 1 coding region and promoter of MECP2 in Rett syndrome and controls. Eur. J. Hum. Genet. 13, 124–126. 10.1038/sj.ejhg.5201270 15367913

[B18] Fehr S. Wilson M. Downs J. Williams S. Murgia A. Sartori S. (2013). The CDKL5 disorder is an independent clinical entity associated with early-onset encephalopathy. Eur. J. Hum. Genet. 21, 266–273. 10.1038/ejhg.2012.156 22872100 PMC3573195

[B19] Fukuda T. Yamashita Y. Nagamitsu S. Miyamoto K. Jin J. J. Ohmori I. (2005). Methyl-CpG binding protein 2 gene (MECP2) variations in Japanese patients with Rett syndrome: pathological mutations and polymorphisms. Brain Dev. 27, 211–217. 10.1016/j.braindev.2004.06.003 15737703

[B20] Gianakopoulos P. J. Zhang Y. Pencea N. Orlic-Milacic M. Mittal K. Windpassinger C. (2012). Mutations in MECP2 exon 1 in classical Rett patients disrupt MECP2_e1 transcription, but not transcription of MECP2_e2. Am. J. Med. Genet. B Neuropsychiatr. Genet. 159B, 210–216. 10.1002/ajmg.b.32015 22213695

[B21] Gilissen C. Hehir-Kwa J. Y. Thung D. T. van de Vorst M. van Bon B. W. Willemsen M. H. (2014). Genome sequencing identifies major causes of severe intellectual disability. Nature 511, 344–347. 10.1038/nature13394 24896178

[B22] Grillo E. Lo Rizzo C. Bianciardi L. Bizzarri V. Baldassarri M. Spiga O. (2013). Revealing the complexity of a monogenic disease: Rett syndrome exome sequencing. PLoS ONE 8:e56599. 10.1371/journal.pone.0056599 23468869 PMC3585308

[B23] Guy J. Gan J. Selfridge J. Cobb S. Bird A. (2007). Reversal of neurological defects in a mouse model of Rett syndrome. Science 315, 1143–1147. 10.1126/science.1138389 17289941 PMC7610836

[B24] Hagberg B. Aicardi J. Dias K. Ramos O. (1983). A progressive syndrome of autism, dementia, ataxia, and loss of purposeful hand use in girls: Rett’s syndrome: report of 35 cases. Ann. Neurol. 14, 471–479. 10.1002/ana.410140412 6638958

[B25] Hayflick S. J. Kruer M. C. Gregory A. Haack T. B. Kurian M. A. Houlden H. H. (2013). beta-Propeller protein-associated neurodegeneration: a new X-linked dominant disorder with brain iron accumulation. Brain 136, 1708–1717. 10.1093/brain/awt095 23687123 PMC3673459

[B26] Kato T. Morisada N. Nagase H. Nishiyama M. Toyoshima D. Nakagawa T. (2015). Somatic mosaicism of a CDKL5 mutation identified by next-generation sequencing. Brain Dev. 10.1016/j.braindev.2015.03.002 25819767

[B27] Kishi N. Macklis J. D. (2004). MECP2 is progressively expressed in post-migratory neurons and is involved in neuronal maturation rather than cell fate decisions. Mol. Cell. Neurosci. 27, 306–321. 10.1016/j.mcn.2004.07.006 15519245

[B28] Klauck S. M. Lindsay S. Beyer K. S. Splitt M. Burn J. Poustka A. (2002). A mutation hot spot for nonspecific X-linked mental retardation in the MECP2 gene causes the Ppm-X syndrome. Am. J. Hum. Genet. 70, 1034–1037. 10.1086/339553 11885030 PMC379098

[B29] Kriaucionis S. Bird A. (2004). The major form of MeCP2 has a novel N-terminus generated by alternative splicing. Nucleic Acids Res. 32, 1818–1823. 10.1093/nar/gkh349 15034150 PMC390342

[B30] Lonetti G. Angelucci A. Morando L. Boggio E. M. Giustetto M. Pizzorusso T. (2010). Early environmental enrichment moderates the behavioral and synaptic phenotype of MeCP2 null mice. Biol. Psychiatry 67, 657–665. 10.1016/j.biopsych.2009.12.022 20172507

[B31] Mnatzakanian G. N. Lohi H. Munteanu I. Alfred S. E. Yamada T. Macleod P. J. (2004). A previously unidentified MECP2 open reading frame defines a new protein isoform relevant to Rett syndrome. Nat. Genet. 36, 339–341. 10.1038/ng1327 15034579

[B32] Moretti P. Levenson J. M. Battaglia F. Atkinson R. Teague R. Antalffy B. (2006). Learning and memory and synaptic plasticity are impaired in a mouse model of Rett syndrome. J. Neurosci. 26, 319–327. 10.1523/JNEUROSCI.2623-05.2006 16399702 PMC6674314

[B33] Neul J. L. Fang P. Barrish J. Lane J. Caeg E. B. Smith E. O. (2008). Specific mutations in methyl-CpG-binding protein 2 confer different severity in Rett syndrome. Neurology 70, 1313–1321. 10.1212/01.wnl.0000291011.54508.aa 18337588 PMC2677974

[B34] Neul J. L. Kaufmann W. E. Glaze D. G. Christodoulou J. Clarke A. J. Bahi-Buisson N. (2010). Rett syndrome: revised diagnostic criteria and nomenclature. Ann. Neurol. 68, 944–950. 10.1002/ana.22124 21154482 PMC3058521

[B35] Okamoto N. Ikeda T. Hasegawa T. Yamamoto Y. Kawato K. Komoto T. (2014a). Early manifestations of BPAN in a pediatric patient. Am. J. Med. Genet. A 164A, 3095–3099. 10.1002/ajmg.a.36779 25263061

[B36] Okamoto N. Miya F. Tsunoda T. Kato M. Saitoh S. Yamasaki M. (2014b). Targeted next-generation sequencing in the diagnosis of neurodevelopmental disorders. Clin. Genet. 10.1111/cge.12492 25156961

[B37] Pelka G. J. Watson C. M. Christodoulou J. Tam P. P. (2005). Distinct expression profiles of Mecp2 transcripts with different lengths of 3′UTR in the brain and visceral organs during mouse development. Genomics 85, 441–452. 10.1016/j.ygeno.2004.12.002 15780747

[B38] Percy A. K. (2008). Rett syndrome: recent research progress. J. Child Neurol. 23, 543–549. 10.1177/0883073807309786 18056689

[B39] Petel-Galil Y. Benteer B. Galil Y. P. Zeev B. B. Greenbaum I. Vecsler M. (2006). Comprehensive diagnosis of Rett’s syndrome relying on genetic, epigenetic and expression evidence of deficiency of the methyl-CpG-binding protein 2 gene: study of a cohort of Israeli patients. J. Med. Genet. 43, e56 10.1136/jmg.2006.041285 17142618 PMC2563193

[B40] Philippe C. Amsallem D. Francannet C. Lambert L. Saunier A. Verneau F. (2010). Phenotypic variability in Rett syndrome associated with FOXG1 mutations in females. J. Med. Genet. 47, 59–65. 10.1136/jmg.2009.067355 19564653

[B41] Quenard A. Yilmaz S. Fontaine H. Bienvenu T. Moncla A. des Portes V. (2006). Deleterious mutations in exon 1 of MECP2 in Rett syndrome. Eur. J. Med. Genet. 49, 313–322. 10.1016/j.ejmg.2005.11.002 16829352

[B42] Ravn K. Nielsen J. B. Schwartz M. (2005). Mutations found within exon 1 of MECP2 in Danish patients with Rett syndrome. Clin. Genet. 67, 532–533. 10.1111/j.1399-0004.2005.00444.x 15857422

[B43] Rett A. (1966). [On a unusual brain atrophy syndrome in hyperammonemia in childhood]. Wien. Med. Wochenschr. 116, 723–726. 5300597

[B44] Rosas-Vargas H. Bahi-Buisson N. Philippe C. Nectoux J. Girard B. N’Guyen Morel M. A. (2008). Impairment of CDKL5 nuclear localisation as a cause for severe infantile encephalopathy. J. Med. Genet. 45, 172–178. 10.1136/jmg.2007.053504 17993579

[B45] Rosenfeld J. A. Crolla J. A. Tomkins S. Bader P. Morrow B. Gorski J. (2010). Refinement of causative genes in monosomy 1p36 through clinical and molecular cytogenetic characterization of small interstitial deletions. Am. J. Med. Genet. A 152A, 1951–1959. 10.1002/ajmg.a.33516 20635359

[B46] Saitsu H. Nishimura T. Muramatsu K. Kodera H. Kumada S. Sugai K. (2013). De novo mutations in the autophagy gene WDR45 cause static encephalopathy of childhood with neurodegeneration in adulthood. Nat. Genet. 45, 445–449. 10.1038/ng.2562 23435086

[B47] Saunders C. J. Minassian B. E. Chow E. W. Zhao W. Vincent J. B. (2009). Novel exon 1 mutations in MECP2 implicate isoform MeCP2_e1 in classical Rett syndrome. Am. J. Med. Genet. A 149A, 1019–1023. 10.1002/ajmg.a.32776 19365833

[B48] Saxena A. de Lagarde D. Leonard H. Williamson S. L. Vasudevan V. Christodoulou J. (2006). Lost in translation: translational interference from a recurrent mutation in exon 1 of MECP2. J. Med. Genet. 43, 470–477. 10.1136/jmg.2005.036244 16155192 PMC2593027

[B49] Suter B. Treadwell-Deering D. Zoghbi H. Y. Glaze D. G. Neul J. L. (2014). Brief report: MECP2 mutations in people without Rett syndrome. J. Autism Dev. Disord. 44, 703–711. 10.1007/s10803-013-1902-z 23921973 PMC3880396

[B50] Swanberg S. E. Nagarajan R. P. Peddada S. Yasui D. H. Lasalle J. M. (2009). Reciprocal co-regulation of EGR2 and MECP2 is disrupted in Rett syndrome and autism. Hum. Mol. Genet. 18, 525–534. 10.1093/hmg/ddn380 19000991 PMC2638799

[B51] Watson P. Black G. Ramsden S. Barrow M. Super M. Kerr B. (2001). Angelman syndrome phenotype associated with mutations in MECP2, a gene encoding a methyl CpG binding protein. J. Med. Genet. 38, 224–228. 10.1136/jmg.38.4.224 11283202 PMC1734853

[B52] Williamson S. L. Christodoulou J. (2006). Rett syndrome: new clinical and molecular insights. Eur. J. Hum. Genet. 14, 896–903. 10.1038/sj.ejhg.5201580 16865103

[B53] Windpassinger C. Kroisel P. M. Wagner K. Petek E. (2002). The human gamma-aminobutyric acid A receptor delta (GABRD) gene: molecular characterisation and tissue-specific expression. Gene 292, 25–31. 10.1016/S0378-1119(02)00649-2 12119096

[B54] Zhang L. He J. Jugloff D. G. Eubanks J. H. (2008). The MeCP2-null mouse hippocampus displays altered basal inhibitory rhythms and is prone to hyperexcitability. Hippocampus 18, 294–309. 10.1002/hipo.20389 18058824

